# Clinical features and disease severity in an Iranian population of inpatients with COVID-19

**DOI:** 10.1038/s41598-021-87917-1

**Published:** 2021-04-22

**Authors:** Shima Nabavi, Zahra Javidarabshahi, Abolghasem Allahyari, Mohammad Ramezani, Mohsen Seddigh-Shamsi, Sahar Ravanshad, Mina AkbariRad, Farnoosh Ebrahimzadeh, Shohre Khatami, Maryam Emadzadeh, Neda Saeedian, Ahmadreza Zarifian, Maryam Miri, Fariba Rezaeetalab, Sepide Hejazi, Reza Basiri, Mahnaz Mozdourian

**Affiliations:** 1 Department of Internal Medicine, Faculty of Medicine, Mashhad University of Medical Sciences, Mashhad, Iran; 2 Lung Diseases Research Center, Mashhad University of Medical Sciences, Mashhad, Iran; 3 Department of Hematology-Oncology, Faculty of Medicine, Mashhad University of Medical Sciences, Mashhad, Iran; 4 Student Research Committee, Faculty of Medicine, Mashhad University of Medical Sciences, Mashhad, Iran; 5 Clinical Research Development Unit, Ghaem Hospital, Mashhad University of Medical Sciences, Mashhad, Iran; 6 Kidney Transplantation Complications Research Centre, Mashhad University of Medical Sciences, Mashhad, Iran

**Keywords:** Biomarkers, Diseases, Medical research, Risk factors, Signs and symptoms

## Abstract

Coronavirus disease 2019 (COVID-19) can present with a variety of symptoms. Severity of the disease may be associated with several factors. Here, we review clinical features of COVID-19 inpatients with different severities. This cross-sectional study was performed in Imam Reza hospital, Mashhad, Iran, during February–April 2020. COVID-19 patients with typical computed tomography (CT) patterns and/or positive reverse-transcriptase polymerase chain reaction (RT-PCR) were included. The patients were classified into three groups of moderate, severe, and critical based on disease severity. Demographic, clinical, laboratory, and radiologic findings were collected and compared. P < 0.05 was considered statistically significant. Overall, 200 patients with mean age of 69.75 ± 6.39 years, of whom 82 (41%) were female were studied. Disease was severe/critical in the majority of patients (167, 83.5%). Disease severity was significantly associated with age, malignant comorbidities, dyspnea, nausea/vomiting, confusion, respiratory rate, pulse rate, O_2_ saturation, extent of CT involvement, serum C-reactive protein (CRP), pH, pO_2_, and aspartate transaminase (P < 0.05). Moreover, complications including shock, coagulopathy, acidosis, sepsis, acute respiratory distress syndrome (ARDS), intensive care unit (ICU) admission, and intubation were significantly higher in patients with higher severities (P < 0.05). O_2_ saturation, nausea/vomiting, and extent of lung CT involvement were independent predictors of severe/critical COVID-19 (OR 0.342, 45.93, and 25.48, respectively; P < 0.05). Our results indicate O_2_ saturation, nausea/vomiting, and extent of lung CT involvement as independent predictors of severe COVID-19 conditions. Serum CRP levels and pO_2_ were also considerably higher patients with higher severity and can be used along with other factors to predict severe disease in COVID-19 patients.

## Introduction

In December 2019, a novel coronavirus emerged in Wuhan city of Hubei Province in China^1^. The virus mainly involved the lungs, leading to a severe acute respiratory syndrome; thus it was initially named as severe acute respiratory syndrome coronavirus 2 (SARS-CoV-2). The world health organization (WHO) named the condition as coronavirus disease of 2019 (COVID-19) and announced it as a global health emergency, which soon was recognized a pandemic^2^.

Compared to the two previously known diseases caused by coronaviruses, namely Middle East Respiratory Syndrome (MERS) and Severe Acute Respiratory Syndrome (SARS), COVID-19 has shown higher estimated infectivity rates (3.5 vs. 0.92 and 3 infections per infected persons, respectively) and thus can be potentially more contagious^3–5^. However, the disease appears to be less lethal than was the case in SARS and MERS^6^. It seems that most of COVID-19 cases develop a mild or even asymptomatic disease. Other patients mostly show signs and symptoms of a mild to moderate upper respiratory tract illness. However, some cases develop severe pneumonia that is accompanied by respiratory failure and even death^7,8^.

Previous studies on Chinese population have reported different underlying diseases and demographic factors to be associated with further deterioration of the condition of COVID-19 patients and worse outcomes^8–10^. Old age, smoking, male sex, and underlying diseases such as chronic kidney disease, chronic obstructive pulmonary disease (COPD), and cerebrovascular disease are reportedly associated with higher disease severity^11,12^. Higher levels of serum biomarkers including lactate dehydrogenase (LDH), C-reactive protein (CRP), and D-dimer, as well as decreased blood platelet and lymphocyte counts have also been associated with more lethal conditions^13^. However, most of our knowledge regarding COVID-19 comes from Chinese studies and there is little known about the clinical and paraclinical findings of the patients in other regions. Our study aims to investigate further demographic, clinical, laboratory, and radiologic findings to design a protocol in order to assess the condition, prognosis, and response to treatment of COVID-19 infected inpatients in Iran.

## Results

### Demographic data and comorbid conditions

Overall, 200 patients were enrolled in the study, of whom 118 (59%) were male and 82 (41%) were female. Mean age of the patients was 69.75 ± 6.39 years. Disease was mild/moderate in 33 cases (16.5%), severe in 139 (69.5%), and critical in 28 (14%). There was no significant difference regarding gender between the three groups of patients. Critical cases were significantly older compared with the mild/moderate and severe groups (P = 0.009). However, there was no significant difference in the mean age when comparing the two groups of mild/moderate and severe/critical (P = 0.149). Table 1 compares the demographic data and comorbid conditions in patients with different degrees of disease severity. Among all comorbid conditions, only malignancy had significantly different frequencies between the three groups (P < 0.001).

**Table 1:** Demographic data and comorbid conditions in patients with different disease severities.

Feature	Disease severity			P_1_	P_2_***
	Mild/moderate (N = 33)	Severe (N = 139)	Critical (N = 28)		
**Demographic data**					
Sex (female)	15 (45.5)	55 (39.6)	12 (42.9)	0.569*	0.807
Age (years)	54.87 ± 18.35	57.80 ± 14.80	66.71 ± 16.61	0.149**	**0.009**
**Comorbid conditions**					
DM	6 (18.2)	43 (30.9)	12 (42.9)	0.093*	0.111
IHD	5 (15.2)	22 (15.8)	7 (25.0)	0.757*	0.476
Hypertension	7 (21.2)	40 (28.8)	10 (35.7)	0.310*	0.454
Asthma	0 (0.0)	5 (3.6)	0 (0.0)	0.314*	0.325
Autoimmune disease	1 (3.0)	3 (2.2)	1 (3.6)	> 0.999*	0.888
CKD	0 (0.0)	2 (1.4)	1 (3.6)	0.438*	0.517
Transplantation	1 (3.0)	0 (0.0)	0 (0.0)	0.024*	0.079
COPD	2 (6.1)	13 (9.4)	1 (3.6)	0.653*	0.721
Cerebrovascular disease	0 (0.0)	3 (2.2)	0 (0.0)	0.438*	0.513
CNS disease	0 (0.0)	2 (1.4)	0 (0.0)	0.528*	0.642
Hepatitis	1 (3.0)	0 (0.0)	0 (0.0)	0.024*	0.079
Hypothyroidism	0 (0.0)	2 (1.4)	1 (3.6)	0.438*	0.517
Malignancy	0 (0.0)	3 (2.2)	5 (17.9)	0.199*	** < 0.001**
Smoking	5 (15.2)	14 (10.1)	4 (14.3)	0.822*	0.544
Addiction	0 (0.0)	5 (3.6)	0 (0.0)	0.593*	0.325
Alcohol use	0 (0.0)	1 (0.7)	0 (0.0)	> 0.999*	0.802
**Clinical characteristics of COVID-19**					
Symptomatic period (days)	6.00 ± 6.50	7.09 ± 4.31	6.81 ± 3.54	0.294**	0.555
Hospital stay (days)	6.48 ± 3.94	7.27 ± 3.39	7.56 ± 4.74	0.249**	0.484

*DM* diabetes mellitus, *IHD* ischemic heart disease, *CKD* chronic kidney disease, *COPD* chronic obstructive pulmonary disease, *CNS* central nervous system.

P_1_: Comparison between mild/moderate and severe/critical groups.

P_2_: Comparison between mild/moderate, severe, and critical groups.

Bold values are statistically significant (P < 0.05).

*Chi-square or Fisher’s exact test.

**Mann–Whitney test.

***Kruskal–Wallis test.

### Vital signs, clinical symptoms, and morbidities

Regarding the vital signs, pulse rate, respiratory rate, and oxygen saturation were significantly associated with the severity of disease, both in two-group and three-group comparisons (P < 0.05; Table 2). The frequency of dyspnea was significantly higher in severe/critical group, while nausea/vomiting was significantly more common among the mild/moderate cases (P = 0.023 and 0.016, respectively). Confusion was significantly more common among critical cases, compared to the mild/moderate and severe groups (P = 0.043). Furthermore, most of COVID-19-associated morbidities including shock, sepsis, coagulopathy, acidosis, ARDS, ICU admission, and intubation were significantly more common among the critical cases, compared with mild/moderate and severe groups (P < 0.001).

**Table 2:** Vital signs, clinical symptoms, and morbidities in patients with different disease severities.

Feature	Disease severity			P_1_	P_2_***
	Mild/moderate (N = 33)	Severe (N = 139)	Critical (N = 28)		
**Vital signs**					
Pulse rate	87.12 ± 20.12	94.71 ± 26.64	106.48 ± 26.36	0.003**	** < 0.001**
Respiratory rate	20.12 ± 1.97	26.64 ± 6.87	26.36 ± 6.76	< 0.001**	** < 0.001**
BP (mmHg)	126.71 ± 21.98	126.74 ± 15.42	129.68 ± 23.68	0.478**	0.467
Temperature (°C)	37.63 ± 0.65	37.65 ± 0.66	37.98 ± 0.83	0.742**	0.084
O_2_ saturation (%)	95.48 ± 1.25	87.80 ± 6.37	82.15 ± 10.00	< 0.001**	** < 0.001**
**Symptoms**					
Fever	23 (69.7)	101 (72.7)	19 (67.9)	0.802*	0.849
Dyspnea	24 (72.7)	123 (88.5)	24 (85.7)	0.023*	0.069
Nausea/vomiting	11 (33.3)	22 (15.8)	4 (14.3)	0.016*	0.055
Cough	29 (87.9)	129 (92.8)	25 (89.3)	0.414*	0.595
Diarrhea	8 (24.2)	19 (13.7)	2 (7.1)	0.082*	0.148
Conjunctivitis	0 (0.0)	2 (1.4)	0 (0.0)	0.528*	0.642
Myalgia	16 (48.5)	69 (49.6)	15 (53.6)	0.849*	0.914
Arthralgia	7 (21.2)	18 (12.9)	2 (7.1)	0.156*	0.261
Weakness	16 (48.5)	65 (46.8)	14 (50.0)	0.901*	0.945
Abdominal pain	1 (3.0)	6 (4.3)	0 (0.0)	0.872*	0.519
Seizure	0 (0.0)	1 (0.7)	0 (0.0)	0.656*	0.802
Headache	3 (9.1)	26 (18.7)	2 (7.1)	0.266*	0.164
Sore throat	1 (3.0)	19 (13.7)	2 (7.1)	0.109*	0.167
Chill	10 (30.3)	28 (20.1)	7 (25.0)	0.240*	0.428
Hyposmia	4 (12.1)	11 (7.9)	2 (7.1)	0.414*	0.710
Fatigue	9 (27.3)	40 (28.8)	7 (25.0)	0.919*	0.916
Confusion	0 (0.0)	2 (1.4)	3 (10.7)	0.517*	**0.043**
Rhinorrhea	1 (3.0)	3 (2.2)	0 (0.0)	0.802*	0.681
**Morbidities**					
Shock	0 (0.0)	1 (0.7)	6 (21.4)	0.231*	** < 0.001**
Sepsis	0 (0.0)	1 (0.7)	8 (28.6)	0.172*	** < 0.001**
ARDS	0 (0.0)	1 (0.7)	27 (96.4)	0.011*	** < 0.001**
DHF	0 (0.0)	1 (0.7)	0 (0.0)	> 0.999*	0.802
ATN	0 (0.0)	1 (0.7)	1 (3.6)	> 0.999*	0.288
Coagulopathy	0 (0.0)	0 (0.0)	1 (3.6)	> 0.999*	**0.046**
Acidosis	0 (0.0)	0 (0.0)	1 (3.6)	> 0.999*	**0.046**
ICU admission	0 (0.0)	0 (0.0)	14 (50.0)	0.085*	** < 0.001**
Intubation	0 (0.0)	0 (0.0)	23 (82.1)	0.023*	** < 0.001**

*BP* blood pressure, *ARDS* acute respiratory distress syndrome, *DHF* diastolic heart failure, *ATN* acute tubular necrosis, *ICU* intensive care unit.

P_1_: Comparison between mild/moderate and severe/critical groups.

P_2_: Comparison between mild/moderate, severe, and critical groups.

Bold values are statistically significant (P < 0.05).

*Chi-square or Fisher’s exact test.

**Mann–Whitney test.

***Kruskal–Wallis test.

### Paraclinical data

Table 3 details the paraclinical findings of patients. As shown in the table, CRP, AST, pH, and pO_2_ were significantly associated with disease severity in three-group comparisons (P < 0.05). However, two-group comparison showed that only CRP and pO_2_ were significantly associated with disease severity (P < 0.05; Table 3). Chest CT showed that the extent of both ground-glass and consolidative pulmonary involvements were significantly higher in the critical and/or severe cases, compared with mild/moderate ones, in both two- and three-group comparisons (P < 0.01). Severity of COVID-19 was not significantly associated with pleural effusion and bronchiectasis in chest CT.

**Table 3:** Paraclinical data in patients with different disease severities.

Feature		Disease severity			P_1_	P_2_***
		Mild/moderate (N = 33)	Severe (N = 139)	Critical (N = 28)		
**Laboratory findings**						
Lymphopenia		24 (75.0)	99 (76.2)	19 (70.4)	0.985*	0.818
VBG pH		7.42 ± 0.05	7.41 ± 0.05	7.10 ± 1.29	0.572**	**0.030**
VBG pO_2_ (mmHg)		36.27 ± 9.11	32.21 ± 9.48	31.85 ± 8.51	0.037**	**0.021**
VBG pCO_2_ (mmHg)		39.01 ± 6.81	40.33 ± 7.84	35.12 ± 7.92	0.824**	0.112
VBG pHCO_3_ (mmHg)		25.86 ± 4.05	25.94 ± 5.05	23.14 ± 4.32	0.705**	0.054
AST (IU/L)		57.92 ± 76.53	48.55 ± 74.94	54.42 ± 31.50	0.470**	**0.031**
ALT (IU/L)		59.64 ± 87.35	50.94 ± 77.99	49.78 ± 60.37	0.210**	0.384
LDH (U/L)		560.20 ± 200.34	660.74 ± 434.76	897.70 ± 287.47	0.310**	0.137
CRP (mg/L)		51.64 ± 52.07	86.22 ± 62.33	167.59 ± 146.86	0.003**	**0.001**
**Chest CT findings**						
Consolidation	None	8 (24.2)	36 (25.9)	5 (17.9)	0.009*	**0.002**
	1 lobe	1 (3.0)	3 (2.2)	0 (0.0)		
	2 lobes	21 (63.6)	55 (39.6)	6 (21.4)		
	3 lobes	0 (0.0)	1 (0.7)	2 (7.1)		
	4 lobes	3 (9.1)	44 (31.7)	15 (53.6)		
GGO	None	7 (21.2)	32 (23.0)	9 (32.1)	< 0.001*	** < 0.001**
	1 lobe	3 (9.1)	0 (0.0)	0 (0.0)		
	2 lobes	13 (39.4)	25 (18.0)	6 (3.6)		
	3 lobes	0 (0.0)	4 (2.9)	0 (0.0)		
	4 lobes	10 (30.3)	78 (56.1)	18 (64.3)		
PE		1 (3.0)	8 (5.8)	4 (14.3)	0.376*	0.168
Bronchiectasis		0 (0.0)	1 (0.7)	0 (0.0)	0.656*	0.802

*VBG* venous blood gas, *AST* aspartate aminotransferase, *ALT* alanine aminotransferase, *LDH* lactate dehydrogenase, *CRP* C-reactive protein, *CT* computed tomography, *GGO* ground-glass opacity, *PE* pleural effusion.

P_1_: Comparison between mild/moderate and severe/critical groups.

P_2_: Comparison between mild/moderate, severe, and critical groups.

Bold values are statistically significant (P < 0.05).

*Chi-square or Fisher’s exact test.

**Mann–Whitney test.

***Kruskal–Wallis test.

### Multivariate analyses

Multivariate logistic regression showed that O_2_ saturation, nausea/vomiting, and lung involvement of ≥ 50% in chest CT were independent predictors of severe/critical COVID-19. Whereas, only O2 saturation showed a significant association with severity when exclusively considering critical patients in multivariate analyses (Table 4). However, none of the assessed variables were significantly associated with ICU admission in a multivariate regression model.

**Table 4:** Multivariate regression for prediction of severe COVID-19.

Predictor	Odds ratio	95% confidence interval		P
		Lower bound	Upper bound	
**Severe or critical disease**				
O_2_ saturation	0.342	0.146	0.800	0.013
Nausea/vomiting	45.937	1.513	1395.069	0.028
CT involvement ≥ 50%	25.483	1.148	565.455	0.041
**Critical disease**				
O_2_ saturation	0.906	0.824	0.997	0.043

### Receiver operating characteristic (ROC) curve analyses

Our multivariate analyses have shown the level of O_2_ saturation to be strongly associated with the severity of COVID-19. ROC curve and area under ROC curve (AUC) were used to determine the cut-off points of O_2_ saturation that can identify severe/critical and critical COVID-19.

**Figure 1:**
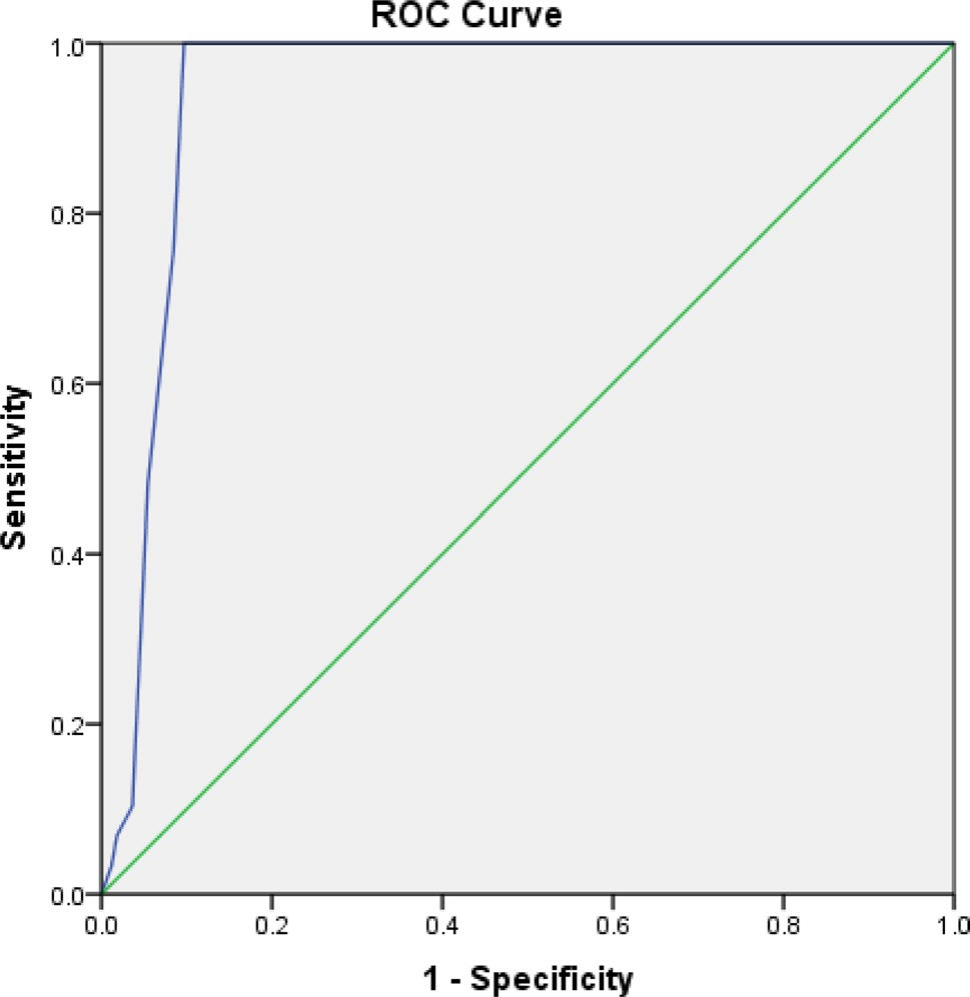
Receiver operating characteristic (ROC) curve of O_2_ saturation for identifying severe/critical COVID-19 infection.

ROC curves showed excellent diagnostic value of O_2_ saturation to identify severe/critical cases (AUC = 0.940, 95% CI 0.907–0.973, P < 0.001; Fig. 1). The best cut-off point was 93.5%, which yielded a sensitivity of 100% and a specificity of 90.3%.

**Figure 2:**
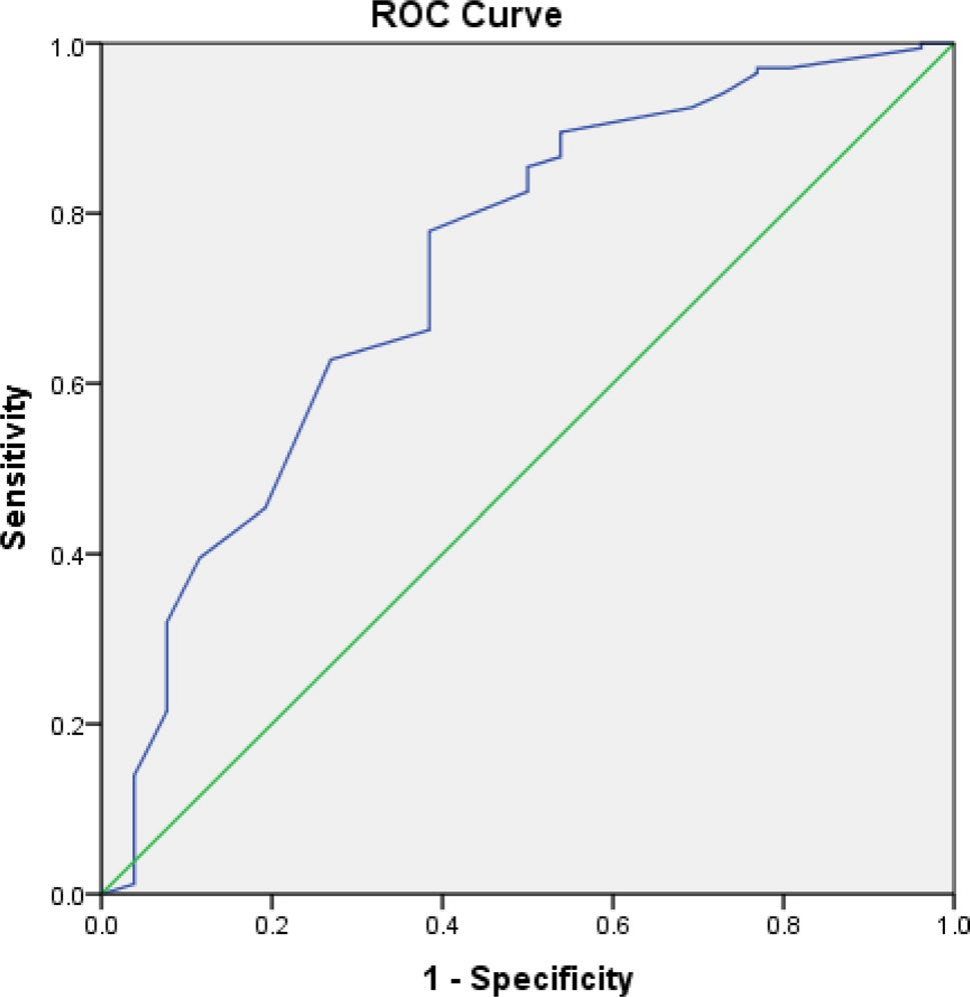
Receiver operating characteristic (ROC) curve of O_2_ saturation for identifying critical COVID-19 infection.

When considering only critical cases of COVID-19, O_2_ saturation showed an acceptable diagnostic value to identify these cases (AUC = 0.735, 95% CI 0.625–0.844, P < 0.001; Fig. 2). The best cut-off point for O_2_ saturation in this regard was 80.5%, which yielded a sensitivity of 89.5% and a specificity of 46.2%.

## Discussion

COVID-19 infection often causes a mild or even asymptomatic disease; however, some patients may proceed to severe and critical condition. Various clinical and paraclinical factors have been associated with higher disease severity^14^. However, most of the studies are from a restricted geographical region and there is a paucity of evidence regarding the determinant factors of poor prognosis in different ethnicities, as the features of COVID-19 might differ in patients with different characteristics. We reviewed the clinical, laboratory, and imaging characteristics of COVID-19 inpatients in a tertiary center in Iran and assessed the factors that might possibly be associated with disease severity.

We found that critical cases of COVID-19 were significantly older compared to patients with lower severity. Malignant comorbidities were found to be considerably higher in critical and severe cases. Among vital signs, pulse rate, respiratory rate, and oxygen saturation were significantly associated with the severity of disease. Among symptoms, dyspnea, confusion, and nausea/vomiting were associated with higher disease severities. COVID-19-associated complications including shock, sepsis, coagulopathy, acidosis, ARDS, ICU admission, and intubation were significantly more common among the critical cases. Paraclinical factors that were associated with higher disease severity were increased CRP and AST, as well as decreased pH and pO_2_. Multivariate analyses showed that O_2_ saturation, nausea/vomiting, and ≥ 50% lung involvement in CT were independent predictors of severe COVID-19. O2 saturation was the sole independent predictor of critical condition in COVID-19 patients. The optimal cut-off values of O_2_ saturation for identifying severe/critical and critical disease were determined at 93.5% and 80.5%, respectively.

In line with the findings of our study, several studies proposed that malignancy is associated with higher COVID-19 severity and poorer outcomes^15–17^. Therefore, it has been proposed that continuing antitumor treatment may further help the outcome of these patients^15^. Specifically, anti-tumor therapy is recommended to be continued in COVID-19 patients with acute leukemia, while for those with low grade malignancies, anti-cancer treatment is better to be postponed during the acute phase of infection^18^.

Although we found higher prevalence of diabetes, hypertension, and cardiovascular diseases among patients with severe disease compared with the mild/moderate group, this difference turned out to be statistically insignificant. This finding is inconsistent with the available literature that indicates a clear link between severity of COVID-19 and comorbidities such as COPD, diabetes, hypertension, and cardiovascular diseases^19–21^. This inconsistency is probably due to the relatively small sample of our study; a larger sample would have yielded statistically significant results, in line with previously reported findings. Moreover, previous studies mostly included Chinese population which may be another factor contributing to the incongruence between their outcomes and the results of our study.

As expected, we observed considerably lower O_2_ saturation and higher values of respiratory and pulse rate in patients with higher severities of the disease. It seems that the pulmonary involvement of COVID-19 and the subsequent respiratory distress, impairs cardiopulmonary functions causing a ventilation-perfusion mismatch^22^, which in turn leads to development of tachypnea and tachycardia. On the other hand, tachycardia can be related to fever in these patients^23^. However, we found no notable difference in the frequency of fever between patients with different disease severities.

AST was also found to be notably higher in severe COVID-19 infection in our patients. It might be hypothesized that the higher rate of hypoxia in more severe stages of the disease may contribute to liver injury and subsequent enzyme release as it is evident with AST release. However, the direct invasion of the virus to hepatocytes can also be proposed as an etiologic factor, which was reported by some studies^24^. Han et al. reported that AST could be an independent risk factor for more severe COVID-19 infections^25^, which was not the case in our multivariate assessments.

Another important finding of our study was the markedly higher level of CRP in patients with severe and critical COVID-19 disease. This factor is reported to be independently related to disease severity; CRP levels > 37.3 mg/L have been reportedly associated with poorer outcomes^25^. In our study, the serum level of CRP showed an incremental increase with the rise in disease severity from mild/moderate to critical. Consistently, Wang et al. reported that higher CRP levels were associated with higher lung involvement and more severe diseases^26^.

Our results indicate that higher severities of COVID-19 are associated with higher rates of serious complications such as shock, sepsis, ARDS, intubation, coagulopathy, and acidosis, which require ICU admission. It is generally believed that most of the COVID-19 cases develop mild to moderate symptoms and do not need hospitalization or ICU admission. However, some of them may need hospitalization and even intensive care. These patients are more likely to develop sepsis, shock, ARDS, and eventually death^27^. Furthermore, despite the usual presence of thrombocytopenia, coagulopathy is predictable in COVID-19 infected patients. Studies have reported elevated levels of D-dimer and higher frequency of thrombotic events in these patients, which might be related to inflammatory processes^28^. Acidosis can be present in some of the COVID-19 patients, which heralds a more severe stage of the disease^7^. In the present study, we found markedly lower pH levels in the venous blood gas (VBG) of patients with critical conditions, compared to other groups. This implies that acidosis is significantly associated with higher severities of the disease.

We found that low O_2_ saturation was the only independent predictor of critical condition and poor prognosis in COVID-19 patients. Higher O_2_ saturation was linked to a one-third lower risk for developing severe/critical disease. In line with our findings, a recent study on 167 patients in Anhui, China, reported that fingertip oxygen saturation and decreased CD4 cell count were the only independent predictors of severe COVID-19^29^.

We also found that the extent and severity of lung involvement on CT scan, as the number of involved lobes with consolidation or ground-glass opacification, was a significant and independent predictor of severe/critical COVID-19 infection. Similarly, Chaganti et al. developed a score for lung involvement that was composed of the number of lobes with consolidation or ground-glass opacification and found that this score was positively correlated with severe stages of COVID-19^30^.

Among all symptoms, nausea/vomiting proved to be an independent predictive factor for severe disease and poorer prognosis. Several studies have indicated that gastrointestinal manifestations, namely nausea and vomiting, are common among COVID-19 patients. However, nausea and vomiting have not been alluded to as risk factors for severe conditions in these patients^31,32^.

A recent systematic review and meta-analysis on 1813 COVID-19 patients showed that dyspnea, COPD, cardiovascular diseases, and hypertension were predictive factors for severe COVID-19 disease and ICU admission^33^. A recent study on 548 patients from Wuhan indicated that older age, comorbid hypertension, high LDH, and D-dimer were significantly associated with higher severity in cases with COVID-19^34^. LDH was also identified as a risk factor for severe disease in another retrospective study of 47 patients from Wuhan, which also indicated lymphocyte count, especially CD3, CD4, and CD8 cells, as a predictive factor for higher severity^35^. Although age was significantly related to disease severity in our study, inconsistent with the mentioned studies, our multivariate analyses did not find significant associations between disease severity and age, comorbid conditions, LDH, and lymphocyte count.

Our study can provide insights into the factors associated with higher risk for developing severe and critical COVID-19 infections in the Iranian population. The present study had some limitations. First of all, we had limited access to RT-PCR testing and could not perform it for all patients. Second, further survival and prognosis analyses was not performed, which may be applicable for further studies. Finally, when compared to the number of infected cases in Iran, our sample is relatively small.

In conclusion, O_2_ saturation, nausea/vomiting, and extent of lung involvement in chest CT can be potential factors that contribute to early prediction of severe and critical conditions in COVID-19 patients. It is therefore recommended to further evaluate the role of these factors in diagnosis and prognosis of patients with COVID-19 in future studies.

## Methods

### Study settings and design

This cross-sectional study was conducted in Imam Reza hospital in Mashhad, the second largest city of Iran, during February–April 2020. With around 900 ward beds and 100 ICU beds, Imam Reza hospital, which is a tertiary center, is recognized as the main public center for COVID-19 patients in Mashhad, Iran. During the COVID-19 pandemic, approximately 400 ward beds and 40 ICU beds were exclusively assigned to COVID-19 inpatients.

### Ethical approval

All enrolled patients provided informed written consent before entrance in to the study. Patients’ data were kept coded without names and confidentiality was observed. The study was performed in accordance with the ethical codes of Helsinki declaration and was approved by the Ethics Committee of Mashhad University of Medical Sciences (approval code: IR.MUMS.REC.1398.308).

### Study population

Patients with confirmed diagnoses of COVID-19 according to a positive RT-PCR and/or typical chest computed tomography (CT) findings were included. The patients were classified based on the severity of disease according to the criteria proposed by WHO^36^, into the following groups:

- *Mild/moderate* with no or mild pneumonia;
- *Severe* with dyspnea (respiratory rate > 30) or hypoxia (O_2_ saturation < 93%);
- *Critical* with respiratory failure, shock, or multi-organ dysfunction.

### Data collection

In order to gather patients’ data, we designed a checklist according to the standards of reporting COVID-19 cases, proposed by WHO^37^. The checklist was discussed in a group of internal medicine specialists and subspecialists using a focus group technique to optimize the list by removing/adding some items.

Demographic data including age and gender, as well as medical and social history were recorded. In addition, clinical symptoms and vital signs were evaluated and recorded. Complications and outcomes including shock, sepsis, acute respiratory distress syndrome (ARDS), diastolic heart failure (DHF), acute tubular necrosis (ATN), intensive care unit (ICU) admission, intubation, coagulopathy, and acidosis were evaluated.

All patients were evaluated for pulmonary involvement using chest CT. The CT scans were then evaluated in terms of presence of abnormalities, mainly ground glass opacities (GGO), consolidation, pleural effusion, and bronchiectasis. The extent of lung involvement on CT scans were also determined based on the number of lobes involved. We considered those with GGO or consolidation in ≥ 3 lobes in the chest CT as having ≥ 50% chest CT involvement.

Blood samples were taken and analyzed for several biomarkers. Laboratory findings including lymphocyte count and serum levels of aspartate aminotransferase (AST), alanine aminotransferase (ALT), LDH, and CRP levels, as well as blood gas analysis were assessed and recorded.

### Statistical analysis

All the analyses were performed using SPSS (version 23 for Windows; IBM Statistics, Chicago, IL). Kolmogorov–Smirnov test was used to assess the normality of data. We made comparisons between all the three groups as well as between mild/moderate and severe/critical groups (merging the severe and critical cases). Chi-square test, Fisher's exact test, Mann–Whitney test, and Kruskal–Wallis test were used to compare data between different subgroups of patients. P < 0.05 was considered statistically significant in all tests.

Multivariate binary logistic regression models were used to assess the factors associated with disease severity and adverse outcomes. Odds ratio (OR) along with 95% confidence interval (95% CI) were used to report the results.

In order to determine the cut-off points of O2 saturation that can differentiate severe/critical and critical disease, ROC curve analyses were performed. Area under the ROC curve was used to confirm the strength of the prediction and optimal cut-off points were selected using the Youden index.
